# A Novel Peptide Isolated from a Phage Display Peptide Library Modeling Antigenic Epitope of DHAV-1 and DHAV-3

**DOI:** 10.3390/vaccines8010121

**Published:** 2020-03-05

**Authors:** Ruihua Zhang, Yupeng Yang, Jingjing Lan, Shaoli Lin, Zhijing Xie, Xiansheng Zhang, Shijin Jiang

**Affiliations:** 1 College of Life Science, Shandong Agricultural University, Taian 271018, China; ruirui041127@126.com (R.Z.); zhangxs@sdau.edu.cn (X.Z.); 2 Department of Preventive Veterinary Medicine, College of Veterinary Medicine, Shandong Agricultural University, Taian 271018, China; 18865485081@163.com (Y.Y.); jjlan1024@163.com (J.L.); lsl1990@umd.edu (S.L.); xiezhj@sdau.edu.cn (Z.X.); 3 Shandong Provincial Key Laboratory of Animal Biotechnology and Disease Control and Prevention, Taian 271018, China

**Keywords:** duck hepatitis A virus, monoclonal antibody, phage display, mimitope, immunization

## Abstract

Duck hepatitis A virus (DHAV), the major pathogen of duck virus hepatitis (DVH), causes severe diseases that threaten the duck industry worldwide. The VP1 protein, a major structural protein of DHAV, is able to induce neutralizing antibody in ducks. The purpose of this study was to identify the antigenic mimotope of DHAV by phage display technology. A monoclonal antibody (mAb) 4E6 against DHAV-1 and DHAV-3 was prepared, and a phage library prepared with the PhD-12 Phage Display Peptide Library Kit was screened with the mAb. A novel peptide, ^1^GLTWKLPPSM^10^ was identified with high affinity to the mAb and could specifically block mAb 4E6 from binding DHAV-1 and DHAV-3. Animal tests confirmed that the immunization of ducklings with the mimotope could inhibit the virus proliferation and protect the ducklings from DVH. In summary, the neutralizing conformational mimotope ^1^GLTWKLPPSM^10^ might be a promising vaccine candidate for the prevention of DHAV infection.

## 1. Introduction

Duck hepatitis A virus (DHAV) causes highly contagious and acute duck viral hepatitis (DVH) to ducklings within 3 weeks old, concomitant with liver necrosis and hemorrhage, and high mortality rate. As one of the pathogens that seriously jeopardize duck industry in Southeast Asia, an outbreak of DHAV infection will usually be followed by great economy losses [1,2]. DHAV has been classified into three serotypes according to results of neutralization tests, namely the classical serotype 1 (DHAV-1) [3,4,5], the serotype isolated in Taiwan only (DHAV-2) [6], and the serotype firstly identified in South Korea (DHAV-3) [7]. No cross-neutralization was found between DHAV-1 and DHAV-2, and limited cross-neutralization has been reported between DHAV-1 and DHAV-3 [6,7,8]. In recent years, the mixed infections of different DHAVs had happened with increasing frequency in eastern Asia [1,2,6], intensifying the difficulty of DVH prevention and control.

DHAV belongs to genus *Avihepatovirus* within the family *Picornaviridae*. Excluding the poly(A) tail, which is irregular in length, the single-stranded positive-sense RNA of the complete DHAV genome is approximately 7700 nucleotides. It is encapsulated in an icosahedral structure, which is assembled with the structural proteins, namely VP0, VP3, and VP1 [3,6]. Among them, VP1 is considered as the external and dominant antigen with several conserved linear epitopes [8,9,10]. 

Different from linear epitopes, conformational epitopes are formed when discontinuous amino acid in the primary protein sequence are folded into close proximity in a high level structure [11,12]. Thus, it is relatively different to map discontinuous epitopes using synthetic peptides or through amino acid mutant selections [13]. As an in vitro screening technique for identifying ligands for proteins and other macromolecules, the phage displayed technology is a critical tool for protein-protein interaction studies [14]. The phage displayed library is comprised of abundant bacteriophages expressing exogenous peptide or protein sequences in their coat proteins, allowing the selection of binding partners for myriad target types by iterative rounds of in vitro panning and amplification, followed by DNA sequencing [13,14]. It offers an efficient alternative to more traditional methods of epitope mapping, especially the conformational ones.

In this study, we identified a conformational epitope, which could be effectively recognized by monoclonal antibody (mAb) 4E6 of DHAV through a 12-mer random peptide phage display library. The results of neutralization assay showed that the mimitope was capable of blocking the virus recognition by mAb. Further animal tests confirmed the peptide ^1^GLTWKLPPSM^10^, which was truncated from the isolated 12-mer peptide ^1^GLTWKLPPSMVH^12^, could inhibit the virus replication in ducklings, suggesting its potential as an epitope vaccine against DVH.

## 2. Materials and Methods

### 2.1. Phage Library, Virus, and Antibodies

The phage library (1.5 × 10^12^ phages, 100 µL) prepared with the Ph.D.-12 Phage Display Peptide Library Kit (cat. no. E8110S; New England Biolabs, USA) was preserved in 50% glycerol in Tris-buffered saline, pH 7.5. The *Escherichia coli* ER2738 strain, which was supplied with the kit, was used to propagate the phages.

The DHAV-1 LY0801 (FJ436047, median embryo lethal dose, ELD_50_ = 10^−5.7^/0.2 mL), DHAV-1 FC16115 (MG515248, ELD_50_ = 10^−5.75^/0.2 mL), DHAV-3 SD1201 strain (KC993890, ELD_50_ = 10^−3.7^/0.2 mL), and DHAV-3 JN1203 strains (KP715480, ELD_50_ = 10^−4.7^/0.2 mL) were individually isolated from the livers of sick cherry valley ducks from Shandong Province, China, and stored in our laboratory [8,15,16].

Using purified DHAV-1 LY0801 strain to immunize BALB/c mice, we finally got a hybridoma cell line secreting IgG1/kappa type mAb, named 4E6, with high affinity to both DHAV-1 and DHAV-3. Horseradish peroxidase (HRP) labeled goat anti-duck antibody, HRP labeled goat anti-rabbit antibody and HRP labeled goat anti-mouse antibody were purchased from KPL (Gaithersburg, MD, USA). Fluorescein 5-Isothiocyanate (FITC) labeled goat anti-mouse antibody, HRP labeled anti-GST (Glutathione S Transferase) mAb and HRP labeled anti-His (histidine) mAb were both purchased from Beyotime Biotechnology (Beijing, China). HRP labeled anti-M13 mAb was purchased from GE Healthcare (München, Germany).

### 2.2. Neutralization Assay for mAb 4E6 against DHAV-1 and DHAV-3

The protein content in ascites was quantitatively determined with a spectrophotometer (DS-11 Series, DeNovix, USA) and then diluted to 2^3^, 2^4^, 2^5^, 2^6^, 2^7^, and 2^8^ in saline. For each embryo, 0.1 mL of diluted mAb 4E6 was incubated with quantitative DHAV-1 or DHAV-3 (100 ELD_50_, 0.1 mL) at 37 °C for 1 h before the injection into embryos via the allantoic route. The control embryos received virus in the absence of mAb incubation. The embryos were allowed to hatch.

### 2.3. Preparation of Viral Antigens

DHAV-1 LY0801 and DHAV-3 SD1201 strains were used as templates to amplify the entire *VP1*, *VP0* and *VP3* genes of DHAV-1 and DHAV-3. The fragments were digested with *EcoR*I and *Xho*I and then inserted into the corresponding region of pFastBacHTB to generate pFastBac-1VP0, pFastBac-1VP3, pFastBac-1VP1, pFastBac-3VP0, pFastBac-3VP3, and pFastBac-3VP1 constructs for subsequent transformation into DH10Bac *E. coli* cells to obtain the recombinant bacmids. Meanwhile, a pFastBacHT-CAT vector was transformed and served as the positive control. According to the baculovirus expression system instructions, two rounds of blue-white screening were designed to isolate the recombinant bacmids, and the positive colonies were re-cultured to obtain b1VP0, b1VP3, b1VP1, b3VP0, b3VP3, and b3VP1 bacmids. 

### 2.4. Indirect Immunofluorescence (IFA) and Western Blotting Assay

*Spodoptera frugiperda* 9 (Sf9) cells were homogenously seeded in 12-well plates and then transfected with 2.0 μg of recombinant bacmid using 5 μL of X-tremeGENE HP DNA Transfection Reagent. Sf9 cells infected with wild baculovirus and mock cells served as positive and negative controls, respectively. At 72 hours post infection (hpi), expressed proteins in cells were analyzed by IFA and western blotting assays.

For IFA, the cells were washed with phosphate-buffered saline (PBS) three times and then fixed with ice-chilled fixative (acetone-methanol, 1:1 v/v) for 10 min at room temperature. The cells infected with different bacmids were individually incubated with mAb 4E6 (1:200) at 37 °C for 1 h. Subsequently, the cells were washed three times and incubated with a secondary antibody, FITC-labeled goat anti-mouse immunoglobulin G (IgG) (1:500), for 1 h in dark conditions. The cells were washed and viewed under an Olympus IMT 2 fluorescence micro-scope (Olympus, Japan). Images were acquired with an identical exposure time and digitally documented.

For western blotting analysis, the Sf9 monolayer underwent three rounds of infection to amplify the bacmids. The cells were washed three times with PBS, and the virus-infected cells were lysed with cell lysis buffer and sonicated on ice. Sodium dodecyl polyacrylamide gel electrophoresis (SDS-PAGE) and western blotting analysis were successively carried out. Proteins were electro-transferred onto the polyvinylidene difluoride (PVDF) membranes (Millipore, Bedford, MA, USA). The membranes were blocked with 1% bovine serum albumin (BSA) in PBS and washed with PBS containing 0.01% phosphate-buffered saline Tween-20 (PBST), followed by incubation with the anti-His mAb or mAb 4E6 at 37 °C for 2 h. The membranes were washed three times with PBST and then incubated with HRP-conjugated goat anti-mouse IgG (1:1000) for 1 h at 37 °C. The membranes were washed three times, and the reaction was visualized with the Diaminobenzidine Tertrahydrochloride (DAB) Horseradish Peroxidase Color Development Kit (Beyotime, China). 

### 2.5. Bio-Panning and Selection of Phages

The mAb 4E6 in ascites was prepared by ammonium sulfate precipitation and then purified using protein G agarose (Roche, Mannheim, Germany). The screening procedure of the phage display library was carried out according to the manufacturer’s instructions. For the first step, the 96-well plate was coated with purified 4E6 (100 µg/mL in 0.1 M NaHCO_3_, pH 8.6) at 4 °C overnight. The plate was washed with TBST (50 mM Tris-HCl, pH7.5, 150 mM NaCl, 0.1% v/v Tween 20), and the coated wells were blocked with 1 mg/mL BSA in 0.1 M NaHCO_3_ buffer. After incubation with the phage library (4 × 10^8^ phages/well) for 1 h at room temperature, the unbound phages were removed by washing ten times with TBST. The bound phages were eluted with 0.2 M glycine-HCl, pH 2.2 containing 1 mg/mL BSA) and immediately neutralized with 1 M Tris-HCl, pH 9.1. The eluted phage was amplified in *E. coli* (ER2738) and titered on LB/IPTG/Xgal plates. The subsequent two rounds of selection were performed similarly to the first step, with the mAb 4E6 concentration reduced to 50 µg/mL and the Tween 20 concentration increased to 0.5%. Individual clones were randomly selected for phage enzyme linked immunosorbent assay (ELISA) and DNA sequencing.

### 2.6. Phage ELISA and DNA Sequencing

A 96-well plate was coated with purified mAb 4E6 (approximately 10 µg/well mAb in 0.1 M NaHCO_3_, pH 8.6) overnight at 4 °C. The plates were washed three times with TBST, followed by blocking with 3% BSA in PBS for 2 h at room temperature. Subsequently, phages (10^10^ pfu/well) were added into the wells, and the plate was incubated for 1 h at room temperature. The plates were washed ten times with TBST, followed by the addition of HRP-conjugated anti-M13 mAb (1:5000) and incubation at 37 °C for 1 h. Subsequently, tetramethylbenzidine (TMB) substrate was added and the plates were incubated at 37 °C for 15 min. The reaction was stopped with 2 M H_2_SO_4_ and the absorbance was measured at 450 nm. Monophages with relatively high absorbance values were selected for sequencing.

Single-stranded DNA was isolated from the monophages according to the instructions supplied with the phage display peptide library kit and sequenced using the 96gIII sequencing primer.

### 2.7. Phage Design and Sequence Analysis

Peptides Pep0 and PepN were synthesized (purity >95%) by GenScript China Inc (Nanjing, Jiangsu, China). The amino acid sequence of Pep0 was ^1^GLTWKLPPSMVH^12^, and the amino acid sequence of PepN, which served as the negative control, was ^1^IGENITNPIKPN^12^. Ten DHAV-1 strains and ten DHAV-3 strains from NCBI were selected for the VP1 sequence alignment with the corresponding epitope region using the Lasergene tool (DNASTAR Inc., Madison, WT, USA). 

### 2.8. Competitive ELISA of Synthetic Peptides

The 96-well plates were coated with 100 µL of DHAV-1, DHAV-3, 1VP1-Sf9 expressed lysate, or 3VP1-Sf9 expressed lysate at an optimal dilution at 4 °C overnight (a preliminary experiment which was designed to determine the most appropriate amount of the antigens used in the competition ELISA test and it was not shown here). The plates were washed three times, followed by blocking with 3% BSA in PBS for 1.5 h at 37 °C. Thereafter, the synthetic peptide Pep0 or PepN (0, 5, 10, 20, 40, 80 and 160 μg/mL) was combined with mAb 4E6 (0.2 μg/mL in PBS) and incubated at 37 °C for 1 h. The peptide/antibody combinations were added to the DHAV-coated 96-well plates and incubated at 37 °C for 1 h. The plates were washed three times with PBST, followed by the addition of HRP-labeled goat anti-mouse IgG (1:4000) and incubation at 37 °C for 1 h. Subsequently, TMB substrate was added and the plates were incubated at 37 °C for 15 min. The reaction was stopped with 2 M H_2_SO_4_ and the absorbance was measured at 450 nm. All the statistical analysis in this study was performed using GraphPad software.

### 2.9. Immunization of Rabbits with Synthetic Pep-Keyhole Limpet Hemocyanin (KLH)

Pep0 and PepN were respectively synthesized and coupled to keyhole limpet hemocyanin (KLH), and named as Pep0-KLH and PepN-KLH. To prepare the polyclonal antibody, rabbits were injected with 200 μg of peptide emulsified in Freund’s complete adjuvant (FCA), boosted 2 weeks later, and re-boosted two additional times at 2-week intervals with 200 μg of peptide emulsified in Freund’s incomplete adjuvant (FIA). Sera were collected at 7 days after the third immunization and detected by ELISA. 

The 96-well plates were respectively coated with the peptide Pep0 (stock concentration, 0.4 μg/mL; 100 μL/well), DHAV-1 and DHAV-3 (concentration was identical to that indicated above) at 4 °C overnight, and then blocked with 3% BSA. Immunized rabbit sera at different dilutions were added to the plates, which were then incubated at 37 °C for 2 h. Antibodies bound to the peptide Pep0, DHAV-1 and DHAV-3 were detected by ELISA with HRP-labeled goat anti-rabbit IgG (1:250) followed by the peroxidase substrate.

### 2.10. Blocking Assay of Pep0 Sera to DHAV

Monolayers of primary duck embryonic hepatocytes (DEH) cells were cultured and infected with DHAV-1 FC16115 and DHAV-3 JN1206 strains respectively. IFA was performed using mAb 4E6 and anti-Pep0 rabbit sera as the primary antibodies and FITC-conjugated goat anti-mouse IgG as the secondary antibody. Images were acquired with an identical exposure time and digitally documented.

DEH cells were uniformly seeded in 12-well plates. Pep0 and PepN at 0, 10, 20, 40, and 80 μg/mL were respectively combined with an optimal concentration of DHAV and then added to the cells. After incubation for 2 h, the mixture was discarded, and the cells were washed. After incubation for 72 h at 37 °C, the cells were collected, and DHAV infection was assessed using real-time quantitative polymerase chain reaction (RT-qPCR) as described in our previous study [17].

### 2.11. Identification of the Essential Amino Acids in the Epitopes

C- or N-terminal deletion mutants of the ^1^GLTWKLPPSMVH^12^ peptide were synthesized (purity >95%) by GenScript China Inc (Table 1), and approximately 1 µg of each mutant was transferred to a nitrocellulose membrane (Millipore, Bedford, MA, USA). Subsequently, anti-Pep0 rabbit sera (1:1000) were incubated with the membrane-bound peptides at 37 °C for 1 h. After washing three times with PBST, the membrane was probed with HRP-conjugated goat anti-rabbit IgG as secondary antibody.

To prepare the GST-Pep fusion protein, the sense and antisense oligonucleotide fragments encoding the corresponding Pep clone, with stop codon, *Hind*III site, and sticky ends of *BamH*I/*Sal*I, were synthesized by Sango Company (Shanghai, China) (Table 1). The synthesized fragments were inserted at the *BamH*I and *Sal*I sites of the pGEX-6P-1 vector to construct the recombinant vectors. The expressed fragments, which were purified with the GST Purification Kit (TaKaRa, Dalian, China), were subjected to 10% SDS-PAGE and finally transferred to nitrocellulose membranes. The membranes were probed with mAb 4E6 (1:1000) as the primary antibody described above.

### 2.12. Immunization of Ducks with Synthetic Pep1-KLH

A total of 90 ducklings were immunized by intramuscular injection with Pep1-KLH (25 μg/duckling) emulsified in FCA on day 1 and with Pep1-KLH (50 μg/duckling) emulsified in FIA on day 7. For the control group, 90 ducklings were immunized with PepN-KLH as described above. Serum samples were collected from live ducklings on day 10, before the challenge with DHAVs. Using HRP-labeled goat anti-duck IgG (1:500), antibodies bound to the peptide Pep0, DHAV-1, and DHAV-3 were detected by ELISA as described above.

For each group, on day 10, 30 ducklings were infected with the fifth generation duck embryo allantoic liquids of the DHAV-1 FC16115 (0.2 mL/duck) by intramuscular and intranasal injections, 30 ducklings were infected with the fifth generation duck embryo allantoic liquids of the DHAV-3 JN1206 (0.2 mL/duck) in the same way, and the rest, 30 ducklings, were infected with both DHAV-1 FC16115 (0.1 mL/duck) and DHAV-3 JN1206 (0.1 mL/duck). The mental state of each duckling was recorded daily for 7 consecutive days and then the ducklings were euthanized. The livers of all ducklings were collected and stored at −80 °C. The RNA viral copy number was determined by RT-qPCR amplification as described in our previous study [17].

## 3. Results

### 3.1. Assessment of Neutralization Activity for mAb 4E6 against DHAV-1 and DHAV-3

In a previous study, we generated a mAb 4E6 with high affinity to both DHAV-1 and DHAV-3. In this study, the neutralizing antibody titer of mAb 4E6 was quantified as 3.54 mg/mL. For each duck embryo, 0.1 mL of diluted mAb 4E6 was used to neutralize 100 median embryo lethal dose (ELD_50_) of DHAV-1 and DHAV-3 (equal volume), respectively. According to the results shown in Table 2, mAb 4E6 neutralized DHAV-1 LY0801 strain with a neutralization titer of 42.2 dilution fold, whereas it neutralized the DHAV-3 SD1201 strain with a neutralization titer of 22.6 dilution fold, which indicated that DHAV-1 was the predominant serotype recognized by mAb 4E6.

### 3.2. A Conformational Epitope in DHAV VP1 Recognized by mAb 4E6

**Figure 1:**
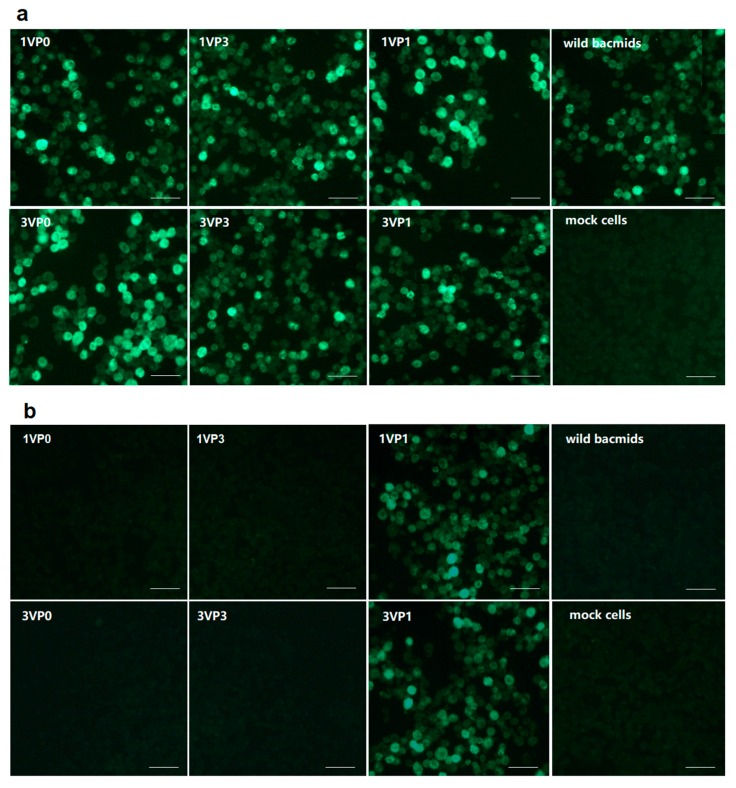
The VP1, VP0, and VP3 proteins of DHAV-1 and DHAV-3 expressed in Sf9 cells were detected by immunofluorescence assay (IFA): (**a**) the Sf9 cells transfected by the different recombinant bacmids (1VP0, 1VP3, 1VP1, 3VP0, 3VP3, 3VP1) were detected by IFA with anti-His mAb, respectively; (**b**) the Sf9 cells transfected by the different recombinant bacmids were detected by IFA with mAb 4E6, respectively. Bars, 100 μm.

Taking DHAV-1 LY0801 and DHAV-3 SD1201 strains as the templates, the entire VP1, VP0, and VP3 genes of DHAV-1 and DHAV-3 were respectively amplified and assembled into baculoviruses. For preliminary analysis of the epitope to mAb 4E6, recombinant protein expressions were caffied out in Sf9 cells infected with corresponding recombinant virus for 72 h. Using mAb 4E6 as the primary antibody, the VP1 proteins of DHAV-1 and DHAV-3 were stained positive, whereas the VP0 and VP3 proteins were stained negative (Figure 1). It indicated that the epitope recognized by 4E6 was located within the VP1 proteins of DHAV-1 and DHAV-3.

**Figure 2:**
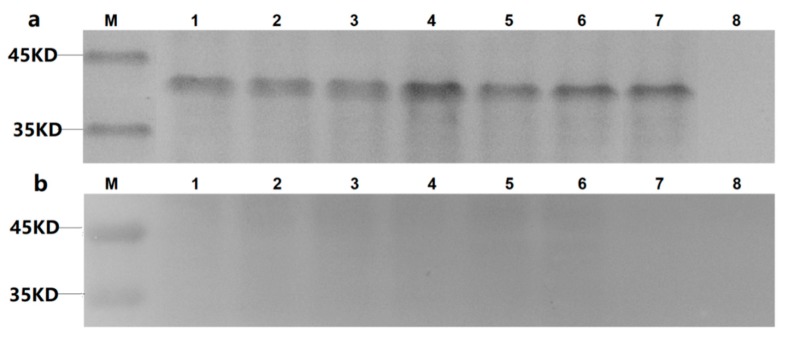
Detection of the recombinant protein expression in Sf9 cells by western blotting assay: (**a**) the Sf9 cells transfected by different recombinant bacmids were detected by western blot with anti-His mAb as the primary antibody; (**b**) the Sf9 cells transfected by different recombinant bacmids were detected by western blot with mAb 4E6 as the primary antibody. Lanes 1–6 represent for cells respectively infected by b1VP0, b1VP3, b1VP1, b3VP0, b3VP3, b3VP1; lane 7 represents for cells infected by wild baculovirus; lane 8 represents for cells un-infected.

Western blotting results showed that VP1, VP0 and VP3 proteins of DHAV-1 and DHAV-3 were successfully expressed but failed to react with mAb 4E6 after linearization (Figure 2), indicating that the epitope recognized by mAb 4E6 was a conformational one.

### 3.3. Identification of the Epitope Recognized with mAb 4E6

To determine the epitope recognized by mAb 4E6, the purified mAb was used for three rounds of phage display 12-mer random peptide library bio-panning. Finally, the phages bound to the mAb with best affinity were obtained and the output to input ratios for each round were as follows: 0.00012%, 0.041%, and 0.96%.

**Figure 3:**
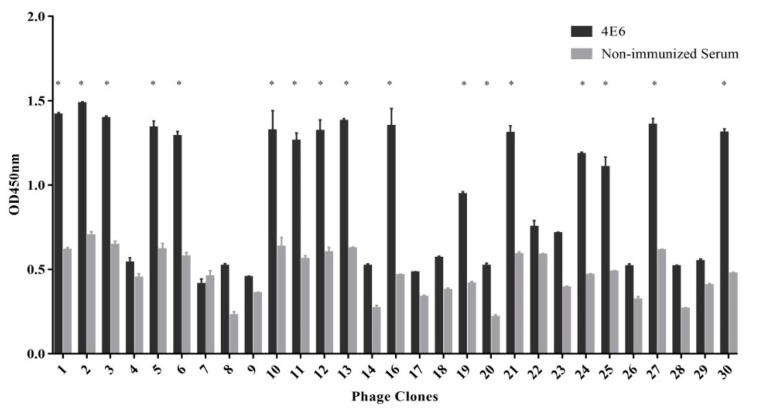
Phage ELISA for the selected phages using mAb 4E6 or non-immunized serum. Three independent assays were performed for each selected phage. *, the phage clones showed specific reactivity with mAb 4E6 (OD_4E6_/OD_non-immunized serum_ > 2.1).

After the third round of bio-panning, 30 phage clones were randomly selected for to assess their reactivity with mAb 4E6 by ELISA. As shown in Figure 3, 17 phage clones showed specific reactivity with mAb 4E6 (OD_4E6_/OD_non-immunized serum_ > 2.1), and the remaining 13 phage clones showed less reactivity with mAb 4E6 (OD_4E6_/OD_non-immunized serum_ < 2.1). The 17 phage clones with high OD values were sequenced and 15 ones contained the concensus sequence ^1^GLTWKLPPSMVH^12^ and the sequence was designated as Pep0. Besides, one of the remaining two sequences, ^1^IGENITNPIKPN^12^, was designated as PepN. To assess the conservation of the eptiope among the DHAVs, we aligned their sequences with the VP1 sequences of 10 DHAV-1 strains and 10 DHAV-3 strains available in GenBank. Overall, the alignment results showed that Pep0 sequence did not exist in the VP1 proteins of DHAV-1 and DHAV-3, and there was no obvious sequence homology.

### 3.4. Synthetic Pep0 Blocked DHAV Binding to 4E6

**Figure 4:**
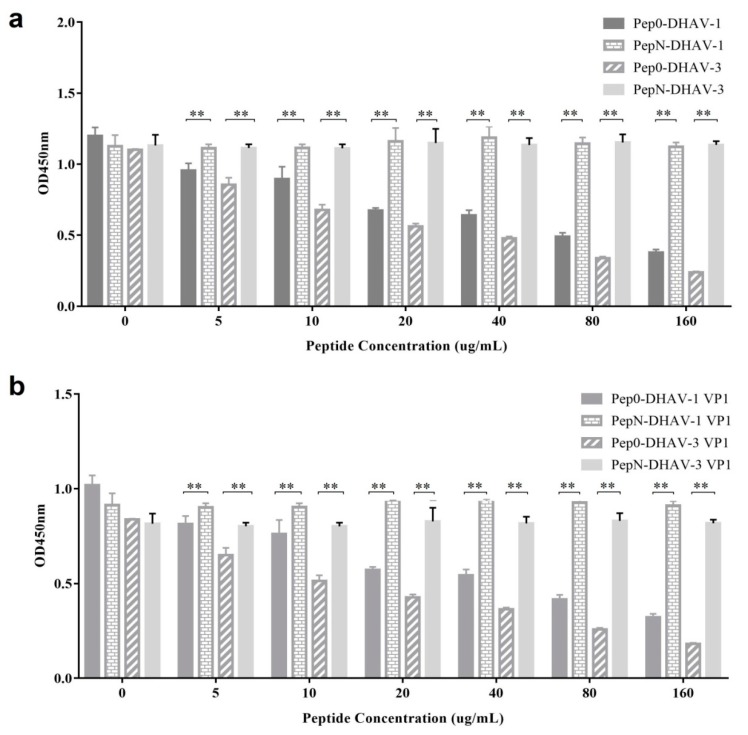
Synthetic peptide Pep0 competitive inhibition of mAb 4E6 to binding DHAVs: (**a**) a competitive ELISA was performed using the antigen peptide as the competitor for the DHAV-1 and DHAV-3; (**b**) a competitive ELISA was performed using the antigen peptide as the competitor for the 1VP1-Sf9 expressed lysate and 3VP1-Sf9 expressed lysate. Values represent three independent experiments with triplicates for each experiment. **, *p* < 0.01.

A competitive ELISA was performed to test whether the synthetic Pep0 prevented mAb 4E6 from binding to DHAV-1 and DHAV-3 virions. Before acting as the primary antibody, a fixed amount of mAb 4E6 was designed to react with Pep0 at different concentrations. The final result showed that the reactivity of the mAb 4E6 with DHAV particles could be inhibited obviously by the synthetic antigen peptide Pep0 in a dose-dependent manner (Figure 4a). However, the control peptide, PepN(^1^IGENITNPIKPN^12^), showed no inhibition reactivity of the mAb 4E6 against DHAV. Moreover, Pep0 could inhibit the reaction of mAb 4E6 with 1VP1-Sf9 expressed lysate and 3VP1-Sf9 expressed lysate (Figure 4b), further suggesting ^1^GLTWKLPPSMVH^12^ was an antigen mimic of VP1.

### 3.5. Rabbits Immunized with Pep0-KLH Produced Anti-DHAV Antibodies

**Figure 5:**
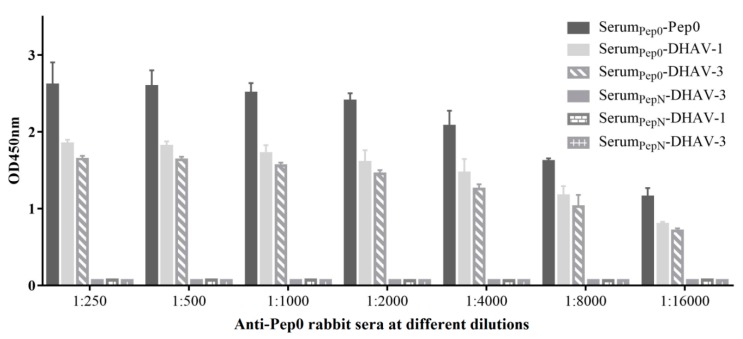
Antibodies bound to the peptide Pep0, DHAV-1, and DHAV-3 in the immunized rabbit sera. The sera were collected from the rabbits at 7 days after the third immunization with Pep0-KLH and tested by ELISA. Values represent three independent experiments with triplicates for each experiment.

Pep0 and PepN were respectively synthesized and coupled to KLH. The immunogenicity of the synthetic peptides was evaluated in rabbits. After the third immunization, the results of the competitive ELISA showed that the rabbits immunized with Pep0-KLH generated peptide-specific antibodies (Figure 5). Although the anti-peptide response was evidently higher than that of DHAV, the antibodies in rabbit sera did cross-react with DHAV-1 and DHAV-3. Additionally, the antibody in the sera showed better reactivity against DHAV-1 than that against DHAV-3. No anti-Pep0 and anti-DHAV responses were observed in PepN-immunized rabbit sera.

### 3.6. Cell Infectivity Assay

**Figure 6:**
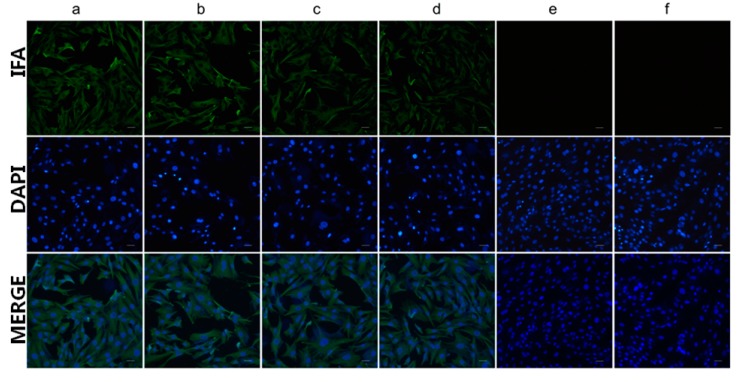
Detection of the DHAV infection in duck embryonic hepatocytes (DEH) cells by IFA: (**a**) the DEH cells infected by DHAV-1 were detected using mAb 4E6; (**b**) the DEH cells infected by DHAV-3 were detected using mAb 4E6; (**c**) the DEH cells infected by DHAV-1 were detected using rabbit anti-Pep1 serum; (**d**) the DEH cells infected by DHAV-3 were detected using rabbit anti-Pep1 serum; (**e**) the DEH cells un-infected were detected using mAb 4E6; (**f**) the DEH cells un-infected were detected using rabbit anti-Pep1 serum. Bars, 50 μm.

To further examine the specificity of mAb 4E6 or anti-Pep0 rabbit sera, DHAV-1 and DHAV-3 were tested by IFA. The results of the IFA showed that both anti-Pep0 rabbit sera and mAb 4E6 could react with DHAV-1- and DHAV-3-infected DEH cells (Figure 6).

**Figure 7:**
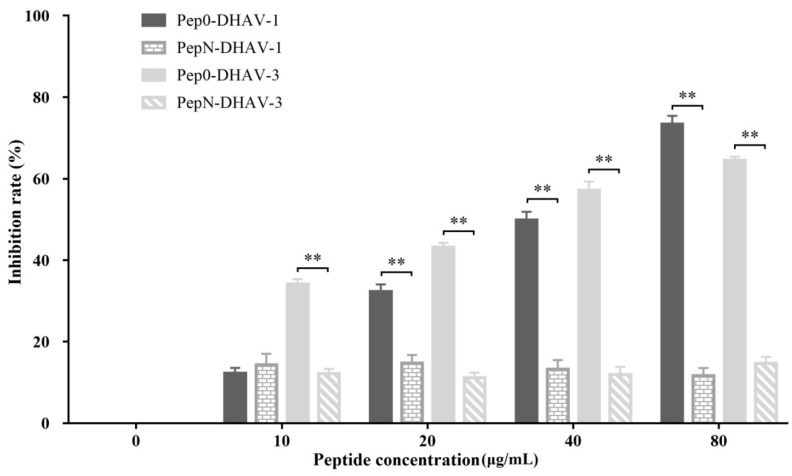
Influence of Pep0 with different concentration on inhibition of DHAV infections. Six-well plates were seeded with equivalent DEH cells and infected with Pep0-DHAV mixtures. The numbers of viral copies were measured using RT-qPCR. The inhibition rate was calculated using the formula (DHAV copies in DHAV singly infection group—DHAV copies in Pep0-DHAV infection group) / DHAV copies in DHAV singly infection group × 100%. Values represent three independent experiments with triplicates for each experiment. **, *p* < 0.01.

As Pep0 could block mAb 4E6 from reacting with DHAV-1 and DHAV-3, we assessed the ability of Pep0 to affect DHAV infection. The results showed that DHAV-1 and DHAV-3 infections were blocked by Pep0 in a dose-dependent manner, whereas an equivalent concentration of PepN had no effect on DHAV-1 and DHAV-3 infections (Figure 7).

### 3.7. Identification of the Essential Amino Acids in Pep0

**Figure 8:**
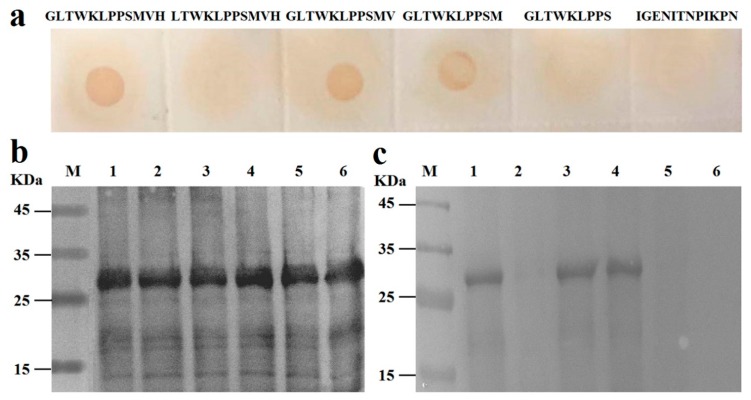
Identification of the essential amino acid of the epitope Pep0: (**a**) Identification of the essential amino acid for the epitope Pep0 based on anti-Pep0 rabbit sera reactivity with the synthesized peptides in the dot blotting assay; (**b**) the reactivity of the expressed mutated peptides to anti-GST (Glutathione S Transferase) mAb; (**c**) the reactivity of the expressed mutated peptides to mAb 4E6. M: Protein marker; lane 1, GST-GLTWKLPPSMVH; lane 2, GST-LTWKLPPSMVH; lane 3, GST-GLTWKLPPSMV; lane 4, GST-GLTWKLPPSM; lane 5, GST-GLTWKLPPS; lane 6, GST negative control.

To define the essential amino acids in Pep0, various fragments were designed and synthesized, corresponding to the roughly mapped epitope ^1^GLTWKLPPSMVH^12^ (Table 2). Dot blotting and western blotting results showed that the deletion of the amino acid ^12^H or ^11^V^12^H of Pep0 at the N-terminus did not affect peptide binding with mAb 4E6 and anti-Pep0 sera, but the deletion of the amino acid ^1^G from the C-terminus or ^10^M^11^V^12^H from the N-terminus completely abolished peptide binding with anti-Pep0 sera (Figure 8). These results indicated that ^1^GLTWKLPPSM^10^ was the essential sequence of the epitope reacting with anti-Pep0 rabbit sera and was designated as Pep1.

### 3.8. Ducks Immunized with Pep1-KLH Could Resist DHAV Infection

**Figure 9:**
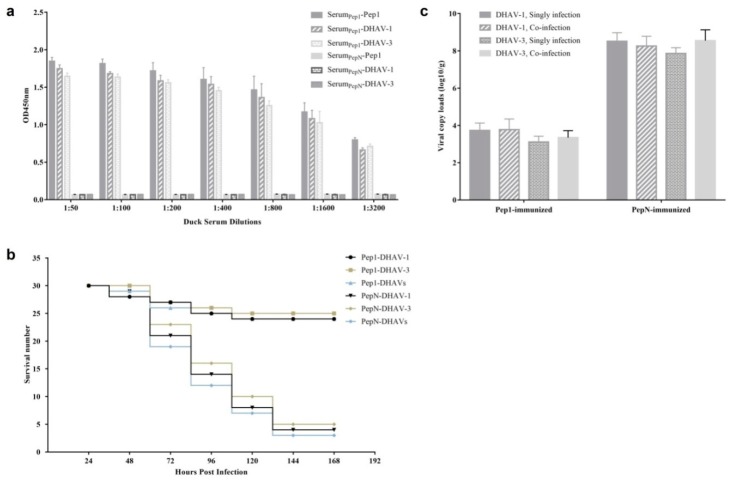
Immune effect analysis of ducklings immunized with synthetic Pep1-KLH: (**a**) Antibodies bound to the peptide Pep0, DHAV-1, and DHAV-3 in the immunized duckling sera were tested by ELISA. Values represent three independent experiments with triplicates for each experiment; (**b**) survival records of the ducklings immunized with Pep1-KLH and PepN-KLH. All of the ducklings’ mental states were recorded daily for seven days; (**c**) the viral loads of DHAV-1 and DHAV-3 in the livers of the singly infected and co-infected ducklings (log10/g). There was no significant difference for the viral loads of DHAV-1 and DHAV-3 between the singly infected and the co-infected ducklings.

Pep1 was synthesized and coupled to KLH for the duck immunization. The ducklings were immunized twice with Pep1-KLH on day 1 and day 7, and challenged with on day 10. Serum samples were collected before the challenge, 3 days after the second immunization. Five samples were randomly selected from every group to detect the antibody by ELISA. The results showed that the ducks immunized with Pep1-KLH generated the DHAV-specific antibodies on day 10, whereas no anti-DHAV antibodies were detected in sera from PepN-KLH immunized ducks (Figure 9a).

In 5 days after virus infection, 24 of 30 DHAV-1-infected ducks, 25 of 30 DHAV-3-infected ducks and 24 of 30 co-infected ducks survived in the Pep1-KLH immunized group, whereas 26 of 30 DHAV-1-infected ducks, 25 of 30 DHAV-3-infected ducks, and 27 of 30 co-infected ducks died with typical symptoms of DVH in the PepN-KLH immunized group (Figure 9b). 

The livers of all ducklings were collected and the viral loads of DHAVs were detected with RT-qPCR. For the dead ducklings in the PepN-KLH immunized groups, the average viral loads of DHAV-1 in the livers were 10^8.51^ copies/g for the singly infected group and 10^8.26^ copies/g for the co-infected group, whereas the average viral loads of DHAV-3 were 10^7.86^ copies/g for the singly infected group and 10^7.55^ copies/g for the co-infected group (Figure 9c). For the live ducks in the Pep1-KLH immunized groups, the average DHAV-1 load in the livers were 10^3.73^ copies/g for the singly infected group and 10^3.77^ copies/g for the co-infected group, whereas the average DHAV-3 loads were 10^3.11^ copies/g for the singly infected group and 10^3.35^ copies/g for the co-infected group. Using an independent-samples t-test, there was no significant difference (*p* > 0.05) in viral loads between the singly infected group and co-infected group. 

## 4. Discussion

For DHAV, a unique member of the *Avihepatovirus* genus of the *Picornaviridae* family, VP1 protein is the most important immunogenic antigen and induces protective neutralizing antibodies in ducks [8,9,10]. Among three genotypes, DHAV-1 and DHAV-3 are considered as the most common continental segregation types [1]. Although possessing VP1 proteins of different lengths (generally 238 and 240 amino acids residues), the results of sequence alignment show a certain degree of amino acid similarity between the two types [4,5]. In previous study, we identified two conserved neutralizing linear B-cell epitope with a mAb 4F8, namely ^75^GEIILT^80^ in DHAV-1 VP1 and ^75^GEVILT^80^ in DHAV-3 VP1 protein [8], further confirming that limited cross neutralization existed between DHAV-1 and DHAV-3.

Epitopes are characterized as linear and conformational epitopes. Different from linear epitopes, which usually contain 6-10 linearized surface amino acids in the target protein, amino acids for the conformational epitopes are discontinuous and difficult to map [18]. In previous reports, several linear epitopes were identified in DHAV with mAb [8,9,10]. To date, there was no conformational epitope being identified in DHAV.

An abundant of phage display peptides constituted the library with all possible amino acid compositions. By screening the library, many mimotopes were identified, and the peptides have been reported to trigger immune responses as successful vaccines [19,20]. Using a nanobody (Nb) DHAV-1 VP1 protein, we previously screened the phage display library of DHAV-1 gene fragments and identified a conserved linear epitope ^174^PAPTST^179^ in DHAV-1 VP1 protein [10]. In this study, purified mAb 4E6 was used to screen a 12-mer phage display peptide library by bio-panning, and the peptide ^1^GLTWKLPPSMVH^12^ was obtained. Further analysis demonstrated that the peptide could specifically block mAb 4E6 from binding DHAV (Figure 4). Moreover, the results of immunization assays demonstrated that the shortened peptide, ^1^GLTWKLPPSM^10^, could trigger an effective immune response to DHAV and inhibit virus infection in vivo (Figure 7). Although sequence alignment showed no homology between the mimotopes and the DHAV VP1 sequence, these results strongly suggested that the peptide was identical to the antigen epitope of DHAV that could be recognized by mAb 4E6.

Mixed infections of DHAV-1 and DHAV-3 are widespread in China’s mainland [21]. In this study, after intramuscular vaccination in ducklings, the synthetic Pep1-KLH displayed the ability of eliciting strong specific antibody against DHAV-1 and DHAV-3 in sera (Figure 9a). The survival rate was 81.1% (73/90) for Pep1-KLH immunized group, whereas 13.3% (12/90) for PepN-KLH immunized group (Figure 9b). The viral loads of DHAV-1 and DHAV-3 in the livers of live ducklings in Pep1-KLH immunized group were significantly lower than those in the livers of dead ducklings in PepN-KLH immunized group (Figure 9c). These results indicated that the immunization of ducklings with the mimotope Pep1 could inhibit replication of both DHAV-1 and DHAV-3 and protect the ducklings from DVH, suggesting that the neutralizing conformational mimotope Pep1 might be a promising vaccine candidate for the prevention of DHAV infection.

In animal models of co-infection, the pathogenicity of one virus could be promoted or suppressed by another virus [22,23]. In current research, the viral loads of DHAV-1 and DHAV-3 in the livers of dead ducklings respectively had no significant difference between single-infected dead ducklings and co-infected ones (Figure 9c), which was consistent with our previous study [17]. In addition, the viral loads of DHAV-1 and DHAV-3 in the livers of live ducklings also respectively had no significant difference between the single infected group or co-infected group (*p >* 0.05). These results indicated that the co-infection of DHAV-1 and DHAV-3 had no effect on the replication and the viral loads of the two viruses *in vivo*.

## 5. Conclusions

In summary, this is the first report on the identification of a cross-reactive mimotope in the VP1 protein of DHAV-1 and DHAV-3. As a neutralizing conformational mimotope of DHAVs, the epitope, ^1^GLTWKLPPSM^10^, would provide new insights for novel vaccine development against DHAV infections.

## Figures and Tables

**Table 1:** Primers used for the epitope identification.

Peptides	Primers	Sequences of Primers and Synthesized Oligonucleotides
**^1^GLTWKLPPSMVH^12^**	F	5’-CGC*GGATCC*GGTCTTACTTGGAAGCTTCCGCCTTCGATGGTGCATTGA*AAGCTT*GTCGACGTC-3’
	R	5’-GACGTCGAC*AAGCTT*TCAATGCACCATCGAAGGCGGAAGCTTCCAAGTAAGACC*GGATCC*GCG-3’
**^2^LTWKLPPSMVH^12^**	F	5’-CGC*GGATCC*CTTACTTGGAAGCTTCCGCCTTCGATGGTGCATTGA*AAGCTT*GTCGACGTC-3’
	R	5’-GACGTCGAC*AAGCTT*TCAATGCACCATCGAAGGCGGAAGCTTCCAAGTAAG*GGATCC*GCG-3’
**^1^GLTWKLPPSMV^11^**	F	5’-CGC*GGATCC*GGTCTTACTTGGAAGCTTCCGCCTTCGATGGTGTGA*AAGCTT*GTCGACGTC-3’
	R	5’-GACGTCGAC*AAGCTT*TCACACCATCGAAGGCGGAAGCTTCCAAGTAAGACC*GGATCC*GCG-3’
**^1^GLTWKLPPSM^10^**	F	5’-CGC*GGATCC*GGTCTTACTTGGAAGCTTCCGCCTTCGATGTGA*AAGCTT*GTCGACGTC-3’
	R	5’-GACGTCGAC*AAGCTT*TCACATCGAAGGCGGAAGCTTCCAAGTAAGACC*GGATCC*GCG-3’
**^1^GLTWKLPPS^9^**	F	5’-CGC*GGATCC*GGTCTTACTTGGAAGCTTCCGCCTTCGTGA*AAGCTT*GTCGACGTC-3’
	R	5’-GACGTCGAC*AAGCTT*TCACATCGAAGGCGGAAGCTTCCAAGTAAGACC*GGATCC*GCG-3’

**Table 2:** Neutralization assay of monoclonal antibody (mAb) 4E6 against Duck hepatitis A virus (DHAV)-1 and DHAV-3.

Virus	Statistical Indicators	Control Group	4E6 Dilution					
			2^−3^	2^−4^	2^−5^	2^−6^	2^−7^	2^−8^
DHAV-1	Number of dead ducks	8	0	0	2	5	8	8
	Protective rate (%)	0	100	100	75	12.5	0	0
DHAV-3	Number of dead ducks	8	0	3	5	7	8	8
	Protective rate (%)	0	100	62.5	37.5	0	0	0
